# Relationship between muscle activation and sagittal knee joint biomechanics in patients with patellofemoral pain syndrome: a cross-sectional study

**DOI:** 10.1186/s43019-025-00259-4

**Published:** 2025-01-24

**Authors:** Byung Sun Choi, Soon Bin Kwon, Sehyeon Jeon, Myeongjun Kim, Yunseo Ku, Du Hyun Ro, Hyuk-Soo Han

**Affiliations:** 1 Department of Orthopedic Surgery, Seoul National University Hospital, Seoul National University College of Medicine, 28 Yeongeon-Dong, Jongno-Gu, Seoul, 03080 Korea; 2 Department of Neurology, Columbia University Irving Medical Center, New York, NY USA; 3 Seoul National University College of Medicine, Seoul, South Korea; 4 Department of Biomedical Engineering, Chungnam National University College of Medicine, Daejeon, South Korea; 5 CONNECTEVE Co., Ltd, Seoul, South Korea

**Keywords:** Patellofemoral pain syndrome, Gait analysis, Electromyography

## Abstract

**Background:**

Patellofemoral pain syndrome (PFPS) is one of the most common conditions affecting the knee joint, yet its pathomechanics remain unclear. The aim of this study was to investigate changes in muscle activation and gait patterns and to analyze the relationship between muscle activation and kinetic gait patterns in patients with PFPS.

**Methods:**

This study included 31 patients with PFPS and 28 healthy volunteers without any symptoms. The sagittal plane motion of the knee joint, representing primary movement of the knee joint, was evaluated to identify changes in gait patterns. Electromyography (EMG) was used to measure muscle activation of vastus medialis (VM), vastus lateralis (VL), semitendinosus (ST), and gastrocnemius (GCM) muscles during gait analysis. Biomechanical features were analyzed during the three phases of the gait cycle; weight acceptance (WA), single limb support (SLS), and swing limb advancement (SLA) (0 ~ 12%, 13 ~ 50%, and 51 ~ 100% of the gait cycle, respectively).

**Results:**

The average knee extension moment (KEM) during WA was lower in the patient group and no significant differences were observed in the knee flexion angle (KFA). With respect to muscle activation, the patient group showed significantly higher muscle activation of the ST muscle in all phases. As the absolute value of the moment increased, the activation of the VM, VL, and ST muscles increased more rapidly in the patient group, especially when KEM was under −1% body weight × height (Bw × Ht) or over 5% Bw × Ht.

**Conclusions:**

Patients with PFPS exhibit elevated muscle activation, particularly in response to changes in the knee extension moment, which is likely a compensatory mechanism to manage knee joint loading during gait. These results highlight altered neuromuscular adaptations in PFPS, suggesting targeted therapies may help improve functional outcomes.

*Level of evidence* III, cross-sectional study

## Introduction

Patellofemoral pain syndrome (PFPS) is characterized by anterior knee pain and is one of the most common conditions affecting the knee joint [1]. Despite its high prevalence [2], the biomechanics and pathomechanics of PFPS remain unclear, which limits effective management strategies [3–5].

Modern gait analysis provides valuable biomechanical and spatiotemporal data, offering insights into PFPS beyond the limitations of conventional anatomical tests [6]. Electromyography (EMG) is commonly used to study neuromuscular conditions and has been applied to PFPS research [7], focusing on parameters such as the vastus medialis oblique (VMO)/vastus lateralis (VL) activation ratio, muscle activation onset [8], reflex response time [9], and medium frequency band (45–96 Hz) [10] to investigate distinct EMG patterns in patients with PFPS. However, none of these studies have explored how differences in muscle activation patterns correlate with the characteristic biomechanics of PFPS.

It is generally accepted that the pathology of patellofemoral pain is related to elevated patellofemoral joint (PFJ) reaction forces [11–13], which is positively correlated with the knee extension moment (KEM) in the sagittal plane [11, 14, 15]. Previous studies have reported that patients with PFPS showed reduced peak KEM during various activities, including level and stair walking [16–18]. Further, other studies reported an increase in KEM after taping or rehabilitation to reduce pain in patients with PFPS [19, 20]. On the basis of these results, it has been suggested that subjects with PFPS reduce KEM to decrease pain and PFJ reaction forces. However, the mechanism of how patients reduce KEM remains unclear. A recent study has shown that the central nervous system (CNS) modulates muscle activation to reduce the load within the joints in a rat model [21], leading to our hypothesis that muscle activation changes to reduce the KEM in patients with PFPS.

This study analyzed the gait and EMG data of subjects with and without PFPS. We hypothesized that muscle activation patterns change to reduce joint loading and pain in patients with PFPS. The purpose of this study was to investigate the relationship between muscle activation patterns and sagittal knee joint biomechanics, specifically focusing on how changes in KEM influence muscle activation during different phases of the gait cycle in patients with PFPS.

## Methods

### Study population

This prospective cohort study was approved by the Institutional Review Board (IRB no. H-1908-011-1052) and was performed in accordance with relevant guidelines and regulations. Written informed consent was obtained from all participants. We included patients on the basis of the following criteria: (1) patellofemoral pain (visual analog scale (VAS) ≥ 4) lasting at least 6 weeks; (2) aggravating pain with knee flexion, climbing stairs, or squatting; and (3) reporting pain during the patellar compression test. A total of 12 subjects were excluded on the basis of the following criteria: (1) age > 35 years; (2) arthritis on X-ray (Kellgren-Lawrence (KL) grade ≥ 2 or patellofemoral joint space ≤ 3 mm); (3) trauma; (4) any prior knee surgery; (5) marked gait impairment that failed gait analysis; (6) any evidence of inflammatory arthritis; and (7) instability or restriction of movement of the knee joint on physical examination [22–24]. For the control group, participants were recruited through advertisements at the hospital. A total of 31 patients and 28 healthy volunteers were included in this study. In the PFPS group, symptom duration ranged from 6 to 60 months, with a mean duration of 23.16 months (standard deviation 19.25 months). Among the patients, 41.94% had bilateral symptoms, while 29.03% had symptoms on the left or right side. Table 1 summarizes participants’ demographic characteristics and spatiotemporal gait features.

**Table 1:** Population characteristics and spatiotemporal gait data of study subjects

	Patients with PFPS (*n* = 31) Mean (SD)	Control group (*n* = 28) Mean (SD)	*P*-value
Sex (male/female)	18/13	20/8	0.284
Age (years)	28.3 (9.1)	23.3 (1.2)	0.004
Height (cm)	168. 1 (8.2)	171.5 (8.3)	0.116
Weight (kg)	64.0 (12.4)	66.0 (9.5)	0.502
Body mass index (kg/m_2_)	22.5 (3.0)	22.4 (2.2)	0.810
Cadence (steps/min)	113.1 (8.1)	114.9 (6.7)	0.372
Gait speed (cm/s)	118.0 (11.8)	128.2 (12.8)	0.002
Stride length (cm)	124.6 (9.5)	133.6 (10.1)	0.001
Step width (cm)	11.6 (2.6)	12.5 (2.9)	0.238

### Data collection

All gait analysis data, including kinetic, kinematic, and spatiotemporal, were collected at the Human Motion Analysis Laboratory. The subjects were asked to walk for a few minutes to get used to the setting. After warming up, an operator with 20 years of experience placed reflective markers on the subjects according to the Helen Hayes marker set. The subjects were asked to walk along a 9-m track. Motion data were collected using 12 charge-coupled device cameras with a three-dimensional optical motion capture system (Motion Analysis Corp., Santa Rosa, CA, USA) at a sampling frequency of 120 Hz. The kinetic data were obtained using two force plates embedded in the floor and normalized to the weight and height of individuals (% Bw × Ht). The kinetic and kinematic data for each joint were averaged after five or six trials of the 9-m walk and then used as study data.

A total of four EMG channels were measured simultaneously along with gait data: vastus medialis (VM), VL, semitendinosus (ST), and gastrocnemius (GCM). The measured EMG signal was bandpass-filtered with a frequency of 20–350 Hz. The filtered signal was rectified, followed by smoothing using the root mean square method over 200 points. The amplitude of smoothed signal was normalized relative to the maximum voluntary contraction (MVC). The MVC of each muscle was measured according to previous studies [25–27]. The temporal axis of both gait and EMG data was normalized from 0% to 100%.

### Statistical analysis

All data extraction and analyses were performed using MATLAB 2018b (MathWorks, Massachusetts) and Microsoft Excel 2010 (Microsoft, Redmond). We analyzed only one leg from each individual to remove statistical dependence caused by multiple observation of single individuals [28]. Data from the leg with the lesion was analyzed for patients with unilateral PFPS, and data from the right leg was analyzed for patients with bilateral PFPS and the control groups. The KFA and KEM were analyzed in detail for each phase of the gait cycle: weight acceptance (WA), single limb support (SLS), and swing limb advancement (SLA). The average, maximum, and minimum values during each cycle were observed to compare the KFA and KEM between patients and the control group. To observe changes in EMG relative to the KEM, KEM values were sorted in increasing order. All four EMG channels were also sorted using the same index of the KEM. The KEM values were rounded to the nearest unit digit, and the corresponding EMG values with the same unit digit index of KEM were averaged. The Student’s *t*-test was performed to compare EMG values between patients and the control group with the same KEM level. The sample size was derived as follows: in the case of knee extension moment, a difference of 15% is usually assumed to be meaningful, and according to previously reported studies, the peak KEM value follows a distribution of 3.2 ± 0.6 (% Bw × Ht) [29, 30]. When the number of study subjects is calculated with an alpha error of 0.05 and a beta error of 0.2, each group requires 26 subjects. For all analyses, *p* < 0.01 was considered to indicate statistical significance.

## Results

**Fig. 1:**
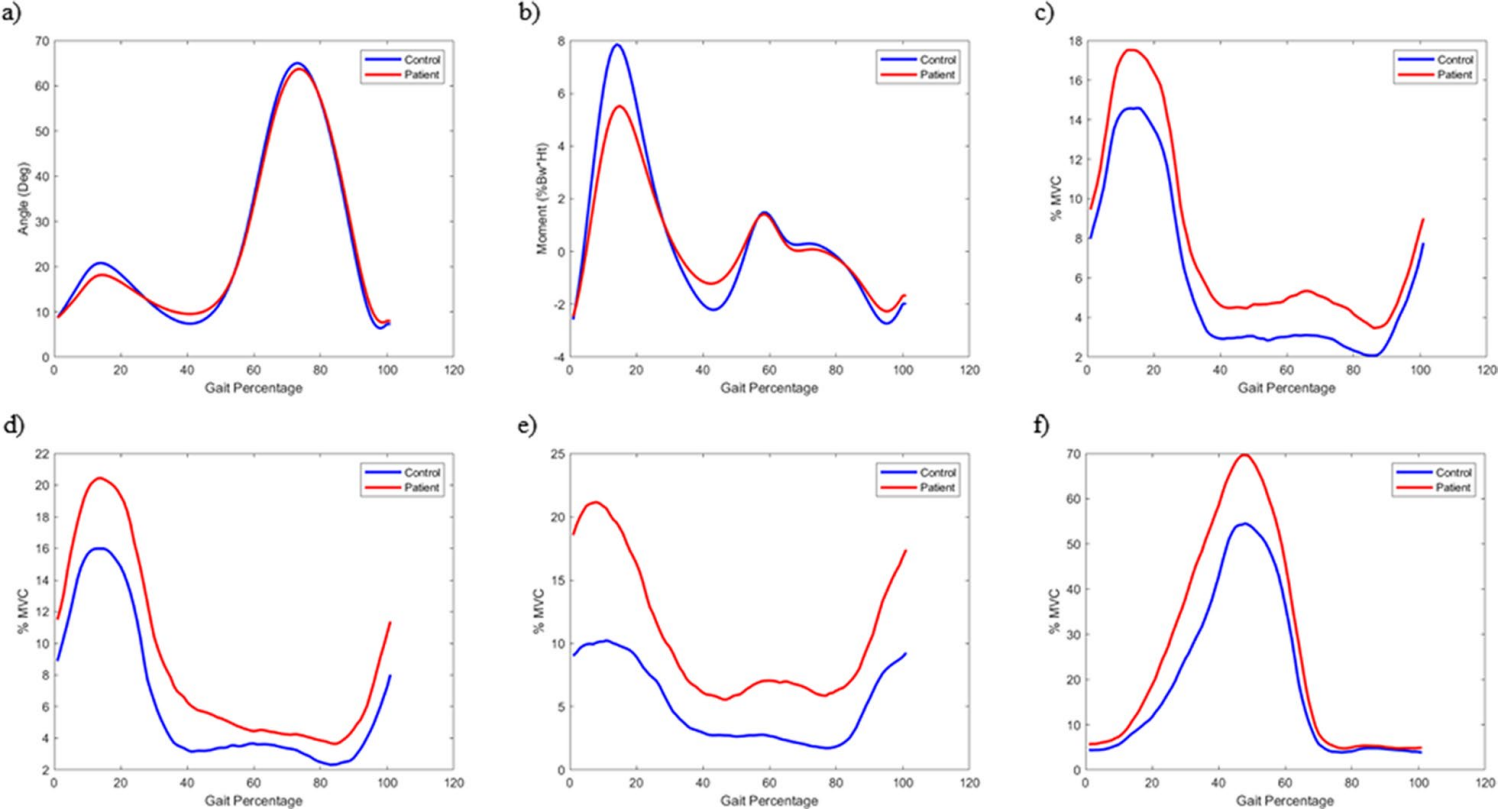
Kinetic, kinematic, and EMG data. All curves represent the mean values of biomechanical features at the point of the gait cycle. Blue represents control; red represents patients. **a** Knee flexion angle; **b** knee extension moment; **c** vastus medilais; **d** vastus lateralis; **e** semitendinosus; **f** gastrocnemius. Knee extension moment was normalized using the weight × height of individuals. All four EMG data were normalized by MVC

The mean and maximum values of KEM during WA were significantly smaller in the patient group (Fig. 1a–b, Table 2).

**Table 2:** Values of knee flexion angle and knee extension moment

			Patient with PFPS (*n* = 31) Mean (SD)	Control group (*n* = 28) Mean (SD)	*P*-value
Knee flexion angle (Deg)	Average	Total	24.83 (2.94)	25.27 (3.32)	0.59
		Weight acceptance	13.46 (3.63)	15.28 (3.71)	0.06
		Single limb support	12.58 (3.39)	12.44 (3.64)	0.88
		Swing limb advancement	37.09 (3.69)	37.62 (3.52)	0.58
	Maximum	Total	63.48 (4.91)	65.26 (4.16)	0.14
		Weight acceptance	18.07 (4.33)	20.71 (4.73)	0.03
		Single limb support	18.51 (4.07)	21.09 (4.59)	0.03
		Swing limb advancement	63.48 (4.91)	65.26 (4.16)	0.14
	Minimum	Total	5.95 (3.68)	4.87 (3.27)	0.24
		Weight acceptance	8.44 (3.57)	8.68 (3.38)	0.79
		Single limb support	8.86 (3.80)	7.13 (3.67)	0.08
		Swing limb advancement	6.80 (4.00)	6.05 (3.64)	0.45
Knee extension moment (% Bw × Ht)	Average	Total	0.29 (0.35)	0.30 (0.28)	0.89
		Weight acceptance^*^	0.99 (0.68)	1.56 (0.62)	< 0.01
		Single limb support	0.70 (0.74)	0.57 (0.69)	0.47
		Swing limb advancement	−0.21 (0.06)	−0.24 (0.06)	0.19
	Maximum	Total^*^	3.39 (1.04)	4.22 (1.04)	< 0.01
		Weight acceptance^*^	3.15 (1.11)	4.08 (1.03)	< 0.01
		Single limb support^*^	3.37 (1.09)	4.20 (1.05)	< 0.01
		Swing limb advancement	0.87 (0.26)	0.83 (0.18)	0.53
	Minimum	Total	−1.52 (0.38)	−1.75 (0.44)	0.03
		Weight acceptance	−1.36 (0.34)	−1.34 (0.39)	0.89
		Single limb support	−0.83 (0.82)	−1.28 (0.80)	0.04
		Swing limb advancement^*^	−1.29 (0.26)	−1.45 (0.18)	< 0.01

*p < 0.01

During SLS, the minimum value was significantly higher in the patient group, and the maximum value was significantly lower. During SLA, the minimum value was significantly higher in the patient group. However, there were no significant differences between the control and patient groups for KFA.

The average muscle activation of the ST muscle was significantly higher throughout the gait cycle in the patient group (Fig. 1c–f, Table 3).

**Table 3:** Muscle activation

			Patients with PFPS (*n* = 31) Mean (SD)	Control group (*n* = 28) Mean (SD)	*P*-value
VM	Average	Total	7.76 (3.96)	5.81 (4.30)	0.08
		Weight acceptance	14.24 (6.82)	11.99 (10.27)	0.33
		Single limb support	9.26 (4.86)	7.07 (5.49)	0.11
		Swing limb advancement	4.93 (3.05)	3.25 (2.12)	0.02
VL	Average	Total	8.92 (4.64)	6.37 (3.24)	0.02
		Weight acceptance	16.89 (9.83)	13.31 (7.30)	0.12
		Single limb support†	11.29 (5.00)	7.68 (3.79)	< 0.01
		Swing limb advancement	5.04 (3.45)	3.56 (2.41)	0.06
ST	Average	Total‡	10.65 (7.16)	5.13 (3.16)	< 0.001
		Weight acceptance†	20.38 (15.14)	9.86 (6.48)	< 0.01
		Single limb support†	10.14 (7.82)	5.32 (3.61)	< 0.01
		Swing limb advancement‡	8.52 (5.77)	3.76 (2.42)	< 0.001
GCM	Average	Total†	25.89 (8.95)	19.24 (8.81)	< 0.01
		Weight acceptance	6.83 (5.79)	5.21 (4.15)	0.23
		Single limb support†	42.77 (17.98)	30.52 (14.04)	< 0.01
		Swing limb advancement	18.02 (5.68)	14.32 (7.14)	0.03

^*^VM vastus medialis, *VL* vastus lateralis, *ST* semitendinosus, *GCM* gastrocnemius

^†^*p* < 0.01; ‡*p* < 0.001

During SLS, the average muscle activation of the VL and GCM muscles was significantly higher in the patient group. The average muscle activation of the GCM during the entire gait cycle was also significantly higher in the patient group.

**Fig. 2:**
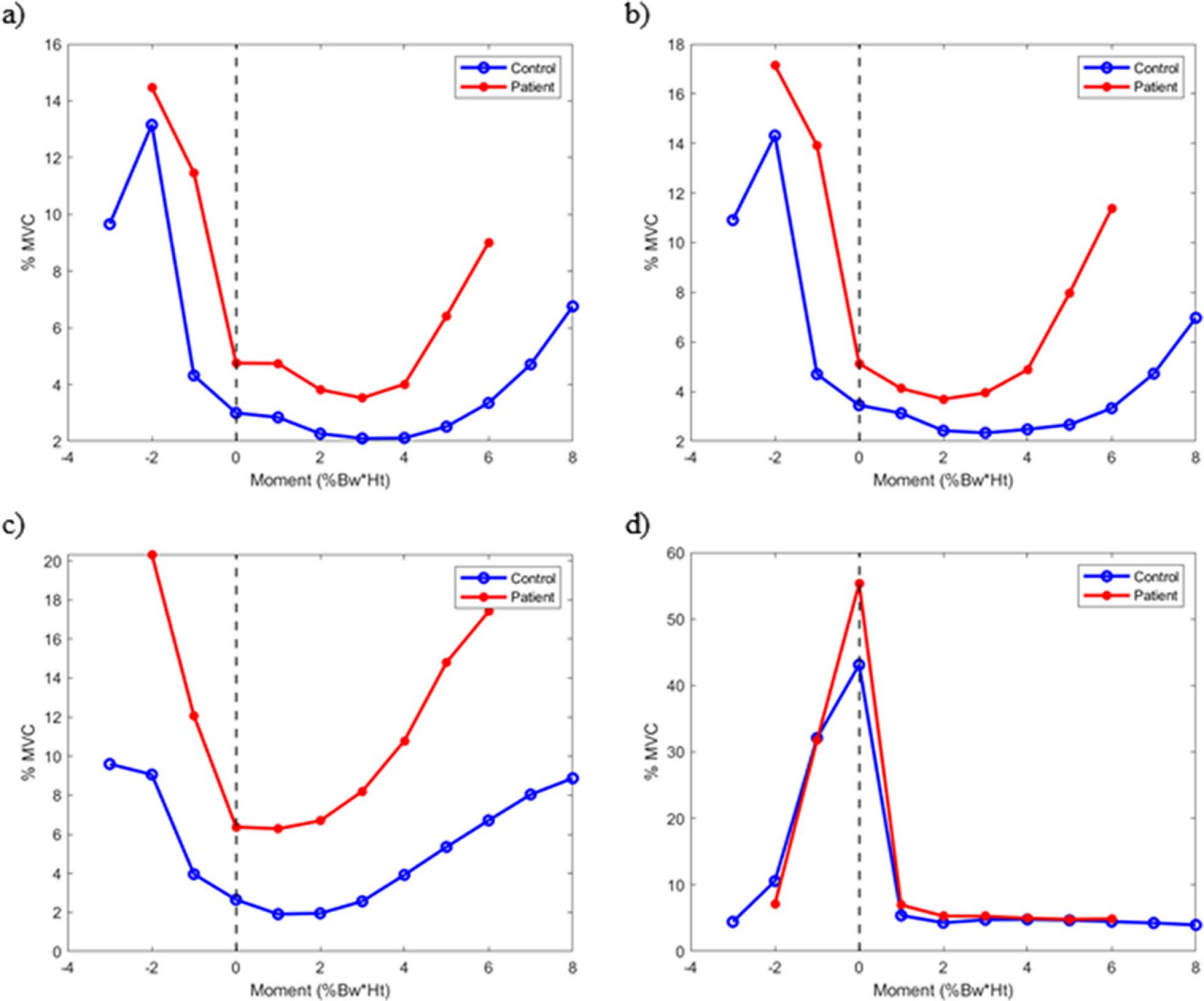
Muscle activation according to the knee extension moment. All points represent the mean values of EMG data in a specific knee extension moment value. The knee extension moment value is determined by rounding it to the nearest ones. Blue represents control; red represents patients. **a** Vastus medilais; **b** vastus lateralis; **c** semitendinosus; **d** gastrocnemius

In the patient group, muscle activation of the VM, VL, and ST generally showed a greater trend compared with the control group across different KEM ranges (Fig. 2, Table 4). However, statistically significant differences were observed only when KEM was under a specific Bw × Ht (VM and VL: −1%, −2%, 5%, 6%; ST: −2%, 5%, 6%; and GCM: −1%, 0% Bw × Ht).

**Table 4:** Muscle activation according to the knee extension moment (KEM). The average of muscle activation was calculated in a specific KEM value range

	KEM (% Bw × Ht)	Control group (*n* = 28)	Patients with PFPS (*n* = 31)	*P*-value
		Mean (SD)	Mean (SD)	
VM (% MVC)	−3	9.66 (8.17)		
	−2‡	13.16 (11.16)	14.47 (7.01)	< 0.001
	−1‡	4.32 (3.2)	11.46 (6.33)	< 0.001
	0	3.00 (2.03)	4.76 (2.96)	0.13
	1	2.84 (1.6)	4.74 (4.79)	0.23
	2	2.27 (1.31)	3.82 (3.42)	0.63
	3	2.10 (1.21)	3.53 (2.51)	0.84
	4	2.12 (1.34)	4.01 (2.02)	0.42
	5‡	2.52 (2.21)	6.42 (2.59)	< 0.001
	6‡	3.35 (4.02)	9.01 (3.38)	< 0.001
	7	4.71 (5.77)		
	8	6.76 (7.2)		
VL (% MVC)	−3	10.91 (6.08)		
	−2‡	14.32 (7.93)	17.14 (9.87)	< 0.001
	−1‡	4.70 (2.69)	13.92 (6.38)	< 0.001
	0	3.45 (2.5)	5.13 (2.92)	0.01
	1	3.13 (2.56)	4.13 (4.19)	0.38
	2	2.43 (2.29)	3.7 (4.14)	0.68
	3	2.34 (2.27)	3.95 (3.96)	0.48
	4	2.48 (2.55)	4.88 (3.89)	0.07
	5‡	2.67 (2.36)	7.98 (5.4)	< 0.001
	6‡	3.33 (2.35)	11.38 (7.85)	< 0.001
	7	4.73 (2.98)		
	8	6.97 (3.95)		
ST (% MVC)	−3	9.59 (5.99)		
	−2‡	9.06 (6.5)	20.32 (15.09)	< 0.001
	−1	3.96 (2.93)	12.07 (9.82)	0.01
	0	2.65 (1.75)	6.38 (4.38)	0.79
	1	1.91 (1.17)	6.28 (5.82)	0.77
	2	1.96 (1.47)	6.69 (6.14)	0.99
	3	2.57 (2.27)	8.20 (6.3)	0.33
	4	3.92 (3.41)	10.79 (7.04)	0.02
	5‡	5.35 (4.57)	14.8 (10.24)	< 0.001
	6‡	6.71 (5.13)	17.4 (12.5)	< 0.001
	7	8.04 (5.58)		
	8	8.87 (5.96)		
GCM (% MVC)	−3	4.44 (4.17)		
	−2	10.57 (6.86)	7.12 (5.78)	0.06
	−1‡	32.10 (15.05)	31.75 (16.37)	< 0.001
	0‡	43.12 (21.13)	55.29 (20.11)	< 0.001
	1	5.40 (3.75)	6.97 (3.44)	0.02
	2	4.31 (3.3)	5.31 (3.59)	0.42
	3	4.75 (4.07)	5.28 (3.77)	0.45
	4	4.80 (4.2)	4.99 (3.8)	0.63
	5	4.68 (4.32)	4.82 (3.87)	0.75
	6	4.47 (4.39)	4.90 (3.98)	0.70
	7	4.26 (4.24)		
	8	3.97 (3.87)		

The KEM value interval was determined by rounding

^*^VM vastus medialis, *VL* vastus lateralis, *ST* semitendinosus, *GCM* gastrocnemius

^‡^*p* < 0.001

## Discussion

This study showed that there were differences present in muscle activity and concomitant gait pattern between patients with PFPS and normal control participants. The sagittal motion, representing the major movement of the knee joint, as indicated by KFA and KEM [31, 32], was analyzed during each phase of the gait cycle. This study further investigated the relationship between muscle activation and KEM by observing changes in four EMG channels. A significant difference was observed only in the KEM, with no significant differences noted in the KFA. Additionally, the differences in EMG activity between the two groups were significant only within specific KEM ranges, reflecting compensatory responses to distinct biomechanical demands.

As shown in Fig. 1 and presented in Table 2, the change in gait pattern was only significant in the kinetic pattern, while no significant difference in the kinematic features was found. The average value of the KEM was significantly different during WA. WA represents 0 ~ 12% of the gait cycle and the main tasks of WA include weight bearing, transferring body weight to the limb, and shock absorption. During the WA phase, the activity of shock-absorbing muscles, including the VL, VM, and ST, showed an increasing trend. However, compared with the control group, these increases were not statistically significant for the VL and VM, indicating minimal differences in muscle activation during level walking owing to its relatively low kinetic demands. In contrast, the ST showed a statistically significant increase in activation in the PFPS group during the entire cycle (Table 3), suggesting its role as a compensatory stabilizer in reducing the KEM by hyperactivating the ST muscle. Kalytczak et al. [33] performed a single leg triple hop test and reported increased EMG activity during the test, with no significant differences in kinematic analysis. There are also reports of reduced KEM during loading response and terminal stance of the gait cycle and increased activity in the VMO and VL muscles in patients with patellofemoral pain compared with healthy control participants [17, 34]. These results suggest that the muscle activity of patients with PFPS alters to reduce the extension moment of the knee joint by increasing muscle activity compared with the control group, without changing the major motion during the gait.

Figure 2, in addition to Table 4, shows that changes in EMG activity are associated with the value of KEM, exhibiting distinct patterns across muscles. For the VM, VL, and ST muscles, a U-shaped relationship was observed, with increased activation at low KEM values (−2 to 0% Bw × Ht) and re-engagement at high KEM values (5–7% Bw × Ht). This pattern suggests an adaptive response aimed at stabilizing the knee joint under specific biomechanical demands [35]. In contrast, the gastrocnemius muscle displayed a mountain-shaped activation pattern, peaking at mid-range KEM values (around 0–3% Bw × Ht), reflecting its primary role in ankle stabilization and propulsion during mid-stance.

Significant differences in muscle activation between the control and PFPS groups were observed primarily at extreme KEM values, highlighting the compensatory neuromuscular strategies employed by patients with PFPS to manage joint loading [36]. Even though the pain of PFPS is aggregated during the extensor activity of the knee joint [37], it is known to not be painful during level walking [38]. The observed alterations in EMG patterns, combined with changes in kinetic gait patterns, indicate that patients with PFPS exert extra effort to stabilize the knee joint and reduce the peak KEM value, even in the absence of pain [39]. The lack of differences in muscle activation at low absolute KEM values reflects the minimal neuromuscular demands during these phases and the absence of compensatory requirements. In contrast, significant differences at high KEM values highlight the adaptive responses in patients with PFPS to manage higher biomechanical loads. While this study suggests a possible role of the CNS in regulating these compensatory mechanisms as proposed in animal models by Barroso et al. [21], further research is needed to directly assess neural control mechanisms in humans with PFPS.

These findings reveal significant alterations in muscle activation patterns and KEM adjustments in patients with PFPS, which may serve as compensatory mechanisms to reduce joint load and alleviate pain [36]. In this study, the PFPS group exhibited notably higher average activation in the VM, VL, and ST across all gait phases compared with the control group. Early increased activation (at −2 to 0% Bw × Ht) may be a compensatory mechanism to preemptively stabilize the knee joint under initial load, while the second rise at higher KEM values (5–7% Bw × Ht) suggests re-engagement of stabilization to control or prevent excessive movement in later phases. These findings support the potential therapeutic value of neuromuscular reeducation and targeted strengthening exercises aimed at optimizing VM, VL, and ST activation [36]. By focusing on balanced activation and controlled KEM adjustments, such interventions could enhance knee stability and reduce excessive joint stress, thereby improving functional outcomes for patients with PFPS.

The specific muscle activation patterns observed in this study are likely influenced by the pathomechanics unique to PFPS. However, patients with knee pain due to other causes may demonstrate different patterns depending on the underlying pathology and the compensatory demands placed on the neuromuscular system. Patients with knee osteoarthritis often show increased co-contraction of quadriceps and hamstring muscles, which is a distinct strategy to enhance joint stability owing to compromised cartilage and structural integrity [36, 40]. Individuals with anterior cruciate ligament (ACL) injuries exhibit altered hamstring activation to compensate for ligamentous instability [41]. To better understand the generalizability of these findings, future studies should compare muscle activation patterns across different knee pathologies.

There are limitations to this study. First, the findings need to be validated with a larger sample size and better consideration of symptom duration variability. Additionally, only four EMG activities were measured, which may limit the comprehensiveness of the analysis. Furthermore, there was an age difference between the patient and control groups, which, although within the young adult range, may have introduced minor variability in the results.

## Conclusions

The findings indicate that patients with PFPS show increased muscle activation as a potential compensatory strategy to reduce knee joint loading during gait. These results highlight altered neuromuscular responses in PFPS, which could inform targeted therapeutic interventions to improve functional outcomes in PFPS management.

## Data Availability

The datasets used and/or analyzed during the current study are available from the corresponding author on reasonable request.
